# The biodiversity theory of allergy: childhood exposure to natural environments and allergy risk

**DOI:** 10.1097/MS9.0000000000004328

**Published:** 2025-11-17

**Authors:** Muhammad Talha, Ubaid Ur Rehman, Mahnoor Fatima, Omar Abdullah Gill, Muneeb Saifullah

**Affiliations:** a Department of Medicine, King Edward Medical University, Lahore, Pakistan; b Department of Medicine, Shaheed Ziaur Rahman Medical College, Rajshahi Medical University, Bogura, Bangladesh

## Introduction

Allergy-related illnesses have increased in numbers in children globally, performing largely as one of the public health problems in recent decades. The prevalence of asthma, allergic rhinitis, eczema, and food allergies in children has increased significantly. This situation runs parallel to rapid urbanization and environmental changes, pushing one to find the environmental influence on immune development^[1]^. The biodiversity theory of allergy, as an extension of the hygiene hypothesis, argues that reduced exposure to different natural environments impairs immune regulation, leading to increased risk of allergy. This minireview intends to provide current evidence that supports this theory, with a special reference to childhood biodiverse environmental exposure and its outcome in allergy^[1,2]^.

## The biodiversity hypothesis

The biodiversity hypothesis builds upon and broadens the earlier hygiene hypothesis, which suggested that the effects of reduced microbial exposure early in life lead to impaired immune regulation. The biodiversity hypothesis posits the loss of environmental microbial diversity as a fundamental cause of rising cases of allergic and inflammatory diseases^[3]^. Thereupon, instead of having concern exclusively for domestic hygiene or avoidance of pathogens, the biodiversity hypothesis acknowledges the need to understand the larger ecological and societal changes embracing urbanization, intensive agriculture, diminishing exposure to natural landscapes, and reduced contact with animals^[4]^.

Interaction with a variety of external microbiota arising from soil, plants, animals, and natural sources of water serves to enrich the human microbiome in different body sites such as skin, gut, and airway, thereby building a strong microbial ecosystem that aids in balanced immune maturation^[3,4]^. These microbes are thought to confer immunological toleration by inducing and maintaining Tregs, therefore favoring response pathways to harmless antigens while tempering excessive inflammatory responses. In contrast, reduced exposure to environmental microorganisms might put children at risk of immune aberrations favoring allergic sensitization and chronic inflammation^[3]^. Hence, the biodiversity hypothesis casts the considerations of allergy prevention not just as matters of individual behaviors or household practices, but also as an ecological-medical issue with implications for urban-planning decisions, public health strategies, and policies for environmental conservation^[3,4]^.

## Evidence in children

A number of epidemiological studies comparing rural with urban children strongly support the hypothesis that exposures to natural environments are associated with a reduction in allergic disease. Meta-analyses have repeatedly documented that children with asthma have, on average, 1.2–1.3 times higher risk in urban than in rural settings. It is remarkable how solid this evidence is, being replicated across different populations and large cohorts containing more than 50 million subjects across the globe^[2]^. Rural children have lower rates of allergic sensitization and clinical allergy when compared with the urban reference group in several studies, including standardized skin prick testing, measurement of allergen-specific IgE from blood samples, and gold-standard food challenge methods^[5]^. Such results further serve to highlight the protective effects conferred by rural and biodiverse settings on immune development and provide cogent evidence that urbanism, through reduced microbial and environmental diversity, is a serious contender in the global rise of allergic disease^[2,5]^.

Green space and biodiversity exposure studies have indicated that the proximity of living areas to forests and agricultural lands has an inverse correlation with atopic sensitization and risk of allergy^[5]^. A study involving cohorts from Finland and Estonia hinted that homes situated amidst more biodiverse land are associated with lower incidences of allergic diseases in children, although the exact characteristics and quality of green space do matter^[6]^. Intervention for biodiversity in daycare was reported to improve immune regulation, evidenced by rises in anti-inflammatory cytokines observed following regular contact with biodiverse soil and vegetation^[6]^. On the other hand, some investigations with mixed results had confounding variables such as pollution, socioeconomic status, and genetically influenced predispositions^[5,6]^.

## Mechanisms

The immunological mechanism at the core of the biodiversity theory emphasizes the interaction of microbes with regulatory T cells (Tregs) in developing immune tolerance. Early exposure to diverse organisms teaches the developing immune system to differentiate harmless antigens from pathogens; this could thus help in controlling exaggerated Th2 responses^[3,7]^. Studies show that diminished environmental biodiversity correlates strongly with decreased microbial diversity in the gut and skin of atopic children, further impairing immune regulation^[3,4]^. The more this balance is disturbed, the greater the allergic sensitization and chronic inflammation are enhanced, linking ecological exposures to immune health^[3,4,7]^.

Treg cells, essential for oral tolerance, are often diminished or dysfunctional in allergy-susceptible infants, tipping the immune balance in favor of sensitivity. A “critical window” refers to the microbial and environmental inputs the infant receives, which instill long-lasting resilience in the immune system^[3]^. Emergent data suggest that limited or delayed microbial factors during this period could confer permanent deficits to immunity. Such changes can predispose children to allergy and potentially predispose them to autoimmune and inflammatory conditions^[4]^. Together, these insights reinforce the significance of biodiversity in protecting lifetime immune health^[3,7]^.

## Public health and ecological implications

Urban planning and the creation of green spaces that promote children’s access to biodiversity could function as strategies to curb the development of allergies and keep their immune system healthy. The Finnish Allergy Programme (2008–2018) put these strategies into practice by means of a strong emphasis on education and contact with nature, leading to a decrease in allergic hospitalizations, health costs, and stabilization in the prevalence of allergies^[8]^. Thus, the conservation of biodiversity is now an act of public health, linking ecological preservation to health benefits for children. There are plans in other European countries and around the world to put similar strategies into practice, proving the scalability of interventions regarding biodiversity^[4]^. When embedded within healthcare and policy frameworks, such strategies could become a sustainable avenue to reversing the growing allergy burden^[4,8]^.

## Limitations and research gaps

The challenge of standardizing measures of biodiversity exposure, whilst disentangling confounders such as air pollution, different lifestyles, and genetic factors, is still an ongoing process^[9]^. Due to a scarcity of randomized controlled trials that directly quantify the effects of biodiversity interventions on allergy incidence, the call for strong causal inference is severely limited, notwithstanding the correlative data available^[4,10]^. Long-term studies employing multiple approaches and integrating ecological perspectives, immunological, and clinical views should be conducted to address the biodiversity hypothesis and its implementation in scalable interventions. The use of additional cutting-edge tools such as microbiome sequencing, wearable exposure sensors, and geospatial biodiversity mapping can be employed to strengthen future studies^[9,10]^. Strong interaction across environmental science, medicine, and public health will be necessary to see theory translated into policy^[10]^.

## Conclusion

This current evidence shows that exposure to biologically diverse natural environments during childhood years acts as a protective factor in reducing allergies by inducing immune regulation mediated by microbiome enrichment and Treg activity. This kind of interrelationship between ecology and medicine needs an integrative approach in urban planning, biodiversity conservation, and public health strategy to dampen the global allergy epidemic.

## Data Availability

Not applicable to this study.

## Notes

M.T.: Writing – original draft and writing – reviewing and editing. U.U.R.: writing – original draft and writing – reviewing and editing. M.F.: Writing – original draft and writing – reviewing and editing. O.A.G.: Writing – original draft and writing – reviewing and editing. M.S.: Writing – original draft and writing – reviewing and editing.
